# Abiotic and Biotic Factors Affecting Resting Spore Formation in the Mite Pathogen *Neozygites floridana*

**DOI:** 10.1155/2013/276168

**Published:** 2013-06-25

**Authors:** Vanessa da Silveira Duarte, Karin Westrum, Ana Elizabete Lopes Ribeiro, Manoel Guedes Corrêa Gondim Junior, Ingeborg Klingen, Italo Delalibera Júnior

**Affiliations:** ^1^Department of Entomology and Acarology, ESALQ/University of São Paulo, 13418-900 Piracicaba, SP, Brazil; ^2^Plant Health and Plant Protection Division, Norwegian Institute for Agricultural and Environmental Research (Bioforsk), 1432 Ås, Norway; ^3^Department of Agronomy, Federal Rural University of Pernambuco, 52171-900 Recife, PE, Brazil

## Abstract

*Neozygites floridana* is an obligate mite pathogenic fungus in the Entomophthoromycota. It has been suggested that resting spores of this fungus are produced as a strategy to survive adverse conditions. In the present study, possible mechanisms involved in the regulation of resting spore formation were investigated in the hosts *Tetranychus urticae* and *Tetranychus evansi*. Abiotic and biotic factors mimicking conditions that we, based on earlier field studies, thought might induce resting spores in temperate and tropical regions were tested with isolates from Norway and Brazil. A total of 42 combinations of conditions were tested, but only one induced the formation of a high number of resting spores in only one isolate. The Brazilian isolate ESALQ1420 produced a large number of resting spores (51.5%) in *T. urticae* at a temperature of 11°C, photoperiod of 10L:14D, and light intensity of 42–46 (**μ**mol m^−2^ s^−1^) on nonsenescent plants (nondiapausing females). Resting spores of the Brazilian *N. floridana* isolate ESALQ1421 were found at very low levels (up to 1.0%). Small percentages of *T. urticae* with resting spores (0–5.0%) were found for the Norwegian isolate NCRI271/04 under the conditions tested. The percentages of resting spores found for the Norwegian isolate in our laboratory studies are similar to the prevalence reported in earlier field studies.

## 1. Introduction

The entomopathogenic fungal genus *Neozygites* belongs to the order Neozygitales in the class Neozygitomycetes in the phylum Entomophthoromycota [1]. Fungi in this genus attack small arthropods such as mealybugs, aphids, thrips, and mites [2]. *Neozygites floridana* (Weiser and Muma) Remaudière and Keller is pathogenic to several species of plant-feeding spider mites [3], and it is an important natural enemy of the two-spotted spider mite, *Tetranychus urticae* Koch, and the red tomato spider mite, *Tetranychus evansi* Baker and Pritchard (Acari: Tetranychidae) [4–6].

For many of the fungal species within the Entomophthoromycota, zygospores and azygospores are important for fungal survival during periods of adverse conditions (e.g., winter, dry season, or host absence), and they are therefore called resting spores [7]. *N. floridana* is an obligate pathogen, and this fungal species may also form resting spores to survive adverse conditions [7–10]. Resting spores of *N. floridana* have been reported in the field in temperate regions in *T. urticae* populations in late summer, fall, and winter [6, 8, 11], and *N. floridana* resting spore prevalences of up to 13.8% were found in *T. urticae* in Norway [8]. Carner [12] suggested that *Neozygites* resting spores were restricted to northern/temperate regions, where the weather is often below freezing during the fall and winter. No field studies on the prevalence of resting spores of *N. floridana* under tropical conditions have been performed, but field studies with *Neozygites tanajoae* Delalibera Jr., Humber, Hajek showed that resting spores of *N. tanajoae* in *Mononychellus tanajoa* Bondar populations were found under tropical conditions in Brazil. Low prevalences of resting spores of *N. tanajoae* in *M. tanajoa* (up to 3.8%) were detected in Brazil by Delalibera Jr. et al. [10], whereas higher prevalences (34–38%) were found by Elliot et al. [9]. However, resting spores of *Neozygites* have not been found in other studies in tropical regions [13–15].

Several factors, such as photoperiod, temperature, host age, inoculum density, and the fungal isolate, may be important for the induction of resting spores in fungi in the Entomophthoromycota [16–21]. For *Zoophthora radicans* (Brefeld) Batko, the resting spore production was negatively correlated with temperature and positively correlated with the relative humidity (RH) and inoculum density [21]. Hajek and Shimazu [20] tested the effects of temperature, photoperiod, and host molting status on resting spore formation by *Entomophaga maimaiga* Humber, Shimazu, and Soper in *Lymantria dispar* (L.); they found that the factor with the greatest impact on the type of spore produced was host age. Resting spore formation was negatively associated with larval molting status; the cadavers of those larvae that molted or exhibited premolt characteristics during the period between infection and death contained fewer resting spores. High levels of fungal inoculum also increased resting spore formation. In a field study, Thomsen and Eilenberg [19] found that *Entomophthora muscae* (Cohn) Fresenius forms resting spores only in female *Delia radicum* (L.) and that the proportion of females with resting spores was negatively correlated with day length. Further, Huang and Feng [18] hypothesized that the resting spore formation of the aphid pathogenic fungus *Pandora nouryi* (Remaudière and Hennebert) Humber depends on the inoculum concentration. Later, Zhou and Feng [17] tested the effects of three parameters on the resting spore formation of *P. nouryi*. Their results suggest that the most important factor for resting spore production is spore density but that temperature and photoperiod are also important. In an even more recent study, Zhou et al. [16] suggested that temperature is the most important factor for the resting spore production of *P. nouryi* in *Myzus persicae* Sulzer under winter field conditions. To the best of our knowledge, no controlled experiments have been conducted to determine which factors are most important for the induction of resting spores in *N. floridana* isolates from temperate or tropical regions. One laboratory study with a Brazilian strain of *N. tanajoae* reported resting spores in 24.2% of *M. tanajoa* individuals under conditions mimicking field conditions at which high prevalences of resting spores were found [9].

Therefore, in the present study, we conducted controlled experiments to identify factors that might be important for the induction of resting spores in *N. floridana* isolates from spider mites from temperate (Norway) and tropical (Brazil) regions. The conditions tested mimic the field conditions under which resting spores have been observed in temperate and tropical regions. Thus, we tested conditions found at the beginning of the dry season in tropical regions and conditions found during the fall and winter in temperate regions. 

*T. urticae* females are known to hibernate during winter [22], and the diapause is induced by short day length [23], but temperature and a lack of nutrition from the host plant may also contribute to the induction of this stage [22]. We hypothesized that infection in diapausing mites might induce resting spore production in *N. floridana*; therefore, we tested diapausing and nondiapausing *T. urticae* as one of the variables in the temperate region treatments.

In the experiment mimicking tropical region conditions, the temperature and RH used were similar to the conditions under which resting spores were found in the field in northeast Brazil, as reported by Delalibera Jr. et al. [10] and Elliot et al. [9]. We also included an experiment where mites were coinfected with two strains of the fungus to test the effect of heterothallism on resting spore production. The nature of *N. floridana* resting spores is still unknown; Humber [24] affirms that there is evidence of heterothallism within the Entomophthoromycota, but Keller [3] suggested that there are indications that *Neozygites fresenii* might be heterothallic.

## 2. Materials and Methods

### 2.1. Experiments Mimicking Temperate Region Conditions

#### 2.1.1. *T. urticae *Culture Reared on Nonsenescent and Senescent Plants

The *T. urticae* used in this culture was collected on the strawberry *Fragaria* × *ananassa* in Ås, Akershus, in southeastern Norway (59° 42′′ N, 10° 44′′ E) in 2003.* T. urticae* were reared on nonsenescent bean plants, *Phaseolus vulgaris* L., in an acclimatized room at 21°C, 60% RH, and L16 : D8. The plants were watered three times per week. Old and weak plants were replaced as needed, usually once a week.

Diapausing *T. urticae* was obtained from old bean plants by maintaining the old plants in a Plexiglas cage in the acclimatized room as described above, but these plants were watered only once per week to stress them and accelerate the process of plant senescence.

#### 2.1.2. *N. floridana* Isolate

Norwegian and Brazilian *N. floridana* isolates were used in the experiments mimicking temperate region conditions. The Norwegian isolate (NCRI271/04) was collected in August 2004, in the same location at which the *T.urticae* was collected, and the Brazilian isolate (ESALQ1420) was collected from *T. urticae* on the jack bean, *Canavalia ensiformis*, in Piracicaba, SP, Brazil (22° 42′ 30′′ S, 47° 38′ 00′′ W).

#### 2.1.3. *N. floridana* Cadaver Production

Leaf discs (1.5 cm diameter) from bean plants were placed underside up on 1.5% water agar in a Petri dish (5 cm in diameter and 2 cm high), and three *N. floridana*-killed *T. urticae* cadavers were placed with their dorsal sides up on the leaf disc. Petri dishes with cadavers on leaf discs were then placed in a plastic box (22 × 16 × 7 cm), covered with aluminum foil to ensure darkness, and incubated at 20°C and 90% RH in a climatic chamber. Cadavers were checked under a compound microscope (80x) after 24 h of incubation, and only the leaf discs with cadavers with good capilliconidia production were used. Thirty uninfected adult *T. urticae* females were then placed on each leaf disc with cadavers for *N. floridana* inoculation. Water was added to the water agar surrounding the leaf disk in the Petri dish to prevent the mites from escaping from the leaf disc. The leaf discs with *T. urticae* were then incubated for 24 h under the conditions described above. Leaf discs containing *N. floridana*-inoculated *T. urticae* were then transferred to uninfested bean plants after 24 h. The mites then walked from the leaf disc onto the bean plant and remained there until they died and mummified. Pods and tendrils were removed to prevent the plant from dangling and allowing the *T. urticae* to crawl off the plant. Leaves that overlapped or grew close together were also cut off to ensure a dry microclimate, keeping the newly mummified cadavers dry and preventing them from sporulating. Plants with *N. floridana*-inoculated *T. urticae* were kept under ambient laboratory conditions at 22–25°C, 20–30% RH, and 24 h light. The dry, nonsporulating cadavers produced on the plant were collected after 7–10 days and kept in small, unbleached cotton cloth pieces in 1.8 mL NUNC Cryo Tubes and stored at 5°C until used in experiments.

#### 2.1.4. Experimental Setup for Abiotic (Light, Temperature) and Biotic (*T. urticae* “Diapause” Condition) Factors

To infect adult *T. urticae* females with the fungus *N. floridana*, we used the protocol described above. Inoculated mites were then transferred with a fine paintbrush onto a bean leaf disc (1.5 cm diameter) placed underside up on 1.5% water agar in 30 mL vials with lids. Twelve holes were made in the lids of the vials with a number 2 insect pin for aeration. At least 60 individual mites were included in each treatment for each isolate. Vials with adult *T. urticae* females were kept under the treatment conditions described in Table 1 until they died of *N. floridana* infection. The two light qualities tested were provided by (1) warm white fluorescent lamps (Philips-Master TL-D 90, referred to as “light quality 1” in this paper) and (2) cool white fluorescent lamps (Mitsubishi-40SW (Ra61), referred to as “light quality 2” in this paper). The effects of a short decrease in temperature were also tested and these treatments were maintained for 4 h at −10, −5, 0, or 5°C during the light period.

Adult *T. urticae* females were evaluated daily during the light period, and dead mites were then mounted in 0.075% Cotton Blue in 50% lactic acid to permit the observation of hyphal bodies and resting spores under a compound microscope (400x). The time of infection lethality (the time from infection to mite death) was calculated for mites with hyphal bodies and for mites with resting spores.

### 2.2. Experiments Mimicking Tropical Region Conditions

#### 2.2.1. *T. urticae* and *T. evansi* Stock Cultures

Both spider mite species (*T. evansi* and *T. urticae*) were collected in Piracicaba, SP, Brazil (22° 42′ 30′′ S, 47° 38′ 00′′ W), and reared on plants maintained in the greenhouse. *T. evansi* was reared on tomato (*Solanum lycopersicum* L.), and *T. urticae* was reared on jack bean (*Canavalia ensiformis* L. (DC)).

#### 2.2.2. *N. floridana* Isolate

The *N. floridana* isolates used in this experiment were collected from *T. urticae* (isolate ESALQ1420) and *T. evansi* (ESALQ1419) on jack bean and tomato, respectively, in Piracicaba, SP, Brazil (22° 42′ 30′′ S, 47° 38′ 00′′ W). A third *N. floridana* isolate (ESALQ1421) was collected from *T. evansi* on tomato, in Recife, PE, Brazil (8° 04′ 03′′ S, 34° 55′ 00′′ W).

#### 2.2.3. *N. floridana *Cadaver Production

Leaf discs (1.2 cm diameter) from jack bean and tomato plants were placed underside up on top of a moist sponge in closed Petri dishes (9 cm diameter). One fungus-killed *T. urticae* or *T. evansi* cadaver was placed on the leaf disc. Petri dishes with cadavers on leaf discs were then placed in a paper box (20 × 20 × 10 cm) to create dark conditions and incubated at 25 ± 2°C and 100% RH in a climatic chamber. Cadavers were checked under a compound microscope (80x) after 24 h of incubation for the production of capilliconidia, and only leaf discs with cadavers with good sporulation and capilliconidia production were used. Twenty uninfected adult *T. urticae* or *T. evansi* females were then transferred with a fine paintbrush to the leaf disc with cadavers for *N. floridana* inoculation. Both spider mites were then incubated for 24 h under the conditions described above. Mites exposed to the sporulating cadavers were then transferred to leaf discs (20 mm diameter) from tomato or jack bean placed underside up on top of moist cotton pads in closed vials (30 mm diameter × 20 mm high) with lids and maintained in a climatic chamber at 25 ± 2°C, 50% RH, and 24 h light. Dry (nonsporulating) cadavers were collected 3–7 days later and kept at −10°C in vials containing silica gel until they were used in the experiment.

#### 2.2.4. Experimental Setup to Test Abiotic (Temperature, RH) and Biotic (Coinfection, Plant Quality, and Mite Age) Factors

To infect spider mites with *N. floridana* isolates (ESALQ1419 or ESALQ1421 to *T. evansi* and ESALQ1420 to *T. urticae*), we used the same protocols described in Section 2.2. (*N. floridana* cadaver production). Vials with *T. urticae* or *T. evansi* were kept under the treatment conditions described in Tables 2 and 3 until they died of *N. floridana* infection.

*Abiotic Factors: Temperature and RH*. Inoculated mites were transferred with a fine paintbrush onto a tomato leaf disc (20 mm diameter) placed underside up on moist cotton pads in vials (30 mm diameter and 20 mm high) closed with lids.

The vials for each treatment were placed on metal supports inside chambers containing saturated salt solutions to achieve the desired humidity. The RH conditions inside the chambers were 50%, 70%, 80%, and 90%, obtained using saturated salt solutions of Mg (NO_3_)_2_6H_2_O, NaCl, KCl, and K_2_SO_4_, respectively, according to Winston and Bates [25]. The RH was measured by a hygrometer at the beginning of the experiment. The chambers were closed with Parafilm to maintain the same RH until the end of the experiment. The chambers were placed in incubators at 32 ± 2°C and 35 ± 2°C and a photoperiod of L12 : D12, and each chamber represented one treatment. After ten days, the mites were checked, and dead and live mites were mounted in Aman Blue for the observation of hyphal bodies and resting spores under a compound microscope (400x). Each treatment and isolate included at least 20 mites, and the experiment was repeated ten times.

*Biotic Factors: Coinfection of Isolates*. In this study, it was investigated whether coinfection with different *N. floridana* isolates would yield mating between individuals of opposite mating types and thus zygospores. This study was conducted by coinfecting *T. evansi* and *T. urticae* hosts with three different *N. floridana* isolates in the following different combinations: *T. evansi* isolate ESALQ1419 × *T. urticae* isolate ESALQ1420 and *T. evansi* isolate ESALQ1419 × *T. evansi* isolate ESALQ1421.

One *N. floridana*-killed *T. evansi* isolate ESALQ1419 cadaver and another *N. floridana*-killed *T. urticae* isolate ESALQ1420 were placed side by side in the centers of the same jack bean or tomato leaf disc (2.0 cm diameter). In the same way, one mummified mite from the *T. evansi* isolate ESALQ1419 and another mummified mite from the *T. evansi* isolate ESALQ1421 were placed side by side in the centers of a tomato leaf disc (2.0 cm diameter). Each leaf disc was placed with the underside up on a moist sponge in a closed Petri dish (9 cm diameter). The closed Petri dishes with cadavers on leaf discs were then placed in a paper box (20 × 20 × 10 cm) to create dark conditions and incubated at 25 ± 2°C and 100% RH. Cadavers were checked under a compound microscope (80x) after 24 h of incubation to select only the leaf discs with cadavers with good capilliconidia production. *Neozygites* conidia were forcibly discharged from the surface of the host forming a halo conidia on the leaf discs around the cadaver. Only leaf discs with at least 300 capilliconidia were used. Twenty adult *T. urticae* or *T. evansi* females were then transferred, with a paintbrush, onto a jack bean or tomato leaf disc, respectively. The Petri dishes were kept at 25 ± 2°C, L12 : D12, and 70% RH for 24 h, and the mites were then transferred onto new leaf discs. After seven days, the mites were checked, and both dead and live mites were mounted in Aman Blue for the observation of hyphal bodies and resting spores under a compound microscope (400x). At least 20 mites were included in each treatment and isolate, and the experiment was repeated ten times.

*Biotic Factors: Host Plant Quality: Leaf Chlorosis and Senescence*. Leaf discs (1.2 cm diameter) from jack bean and tomato with or without chlorosis were placed underside up on a moist sponge in closed Petri dishes (9 cm diameter). Chlorosis was induced by infestation with high densities of *T. urticae* until more than 50% of the green color of the leaves was lost. One *N. floridana*-killed *T. urticae* (ESALQ1420) or *T. evansi* (ESALQ1421) cadaver was placed on jack bean or tomato leaf disc, respectively. Petri dishes were then placed in a paper box to create dark conditions and incubated at 25 ± 2°C and 100% RH. Cadavers were checked under a compound microscope (80x) after 24 h of incubation to ensure good sporulation and production of capilliconidia. Only leaf discs with over 300 capilliconidia were used. Twenty uninfected adult *T. urticae* or *T. evansi* females were then transferred, with a paintbrush, onto each leaf disc with cadavers for *N. floridana* inoculation and incubated for 24 h under the conditions described above. *N. floridana*-inoculated *T. evansi* and *T. urticae* were then transferred to vials (3.0 cm diameter × 2.0 cm high) closed with lids. The vials were incubated at 25 ± 2°C during the light period and at 15 ± 2°C during the dark period (L11 : D13) with 70% RH. When the mites died, they were mounted in Aman Blue to enable the observation of hyphal bodies and resting spores under a compound microscope (400x). Each treatment and isolate included 20 mites, and the experiment was repeated ten times. To test the effect of leaf senescence on *N. floridana* resting spore production, 60 senescent leaves of the nightshade *Solanum americanum* Mill. were collected. These leaves were kept in small cages (11 × 11 × 3.5 cm) with a moist sponge. *N. floridana*-infected adult *T. evansi* females were then placed on these leaves and incubated at 25 ± 2°C, L12 : D12, and 70% RH. Approximately 680 fungus-killed mites were mounted in Aman Blue for the observation of hyphal bodies and resting spores under a compound microscope (400x).

*Biotic Factors—Mite Age*. To test the effect of mite age on *N. floridana* resting spore production, newly hatched larvae and adult *T. urticae *females were inoculated with *N. floridana* isolate ESALQ1420 using the protocol described above. Inoculated *T. urticae *larvae and adults females were transferred with a fine paintbrush onto a jack bean leaf disc (2.0 cm diameter) placed underside up on moist cotton in vials (3.0 cm diameter and 2.0 cm high) closed with lids. The vials were incubated at 25 ± 2°C, 70% RH, and L12 : D12. After seven days, the mites were checked, and dead and live mites were mounted in Aman Blue for observation of hyphal bodies and resting spores under a compound microscope (400x). At least 20 mites were included in each treatment, and the experiment was repeated ten times.

### 2.3. Statistical Analysis

The effects of different abiotic and biotic factors on the percentage of *T. urticae* with resting spores were analyzed with ANOVA after the arcsine transformation of the data. When significant effects were found, post hoc comparisons using Tukey's HSD test were conducted to evaluate the pairwise differences between means (*P* < 0.05). All statistical analyses were carried out in the SAS package (SAS Institute Inc., Cary North Carolina).

## 3. Results

### 3.1. Experiments Mimicking Temperate Region Conditions

A total of 3,106 mites (not including a series of pilot experiments) were tested at 26 different combinations of conditions. However, a significantly higher rate of resting spores, 51.5% (*F* = 20.5, *P* < 0.0001), was found for only one condition: the Brazilian *N. floridana* isolate at 11°C (no temperature drop) with a photoperiod of 10L : 14D, light intensity of 42–46 (*μ*mol m^−2^ s^−1^), and light quality of 1 in nondiapausing *T. urticae* females from nonsenescent plants (Table 1). No significant difference in resting spore production was observed for any of the other combinations of conditions for any of the isolates tested. One combination of conditions resulted in a low level of resting spore production (1.4%) for the Brazilian *N. floridana* isolate in *T. urticae* females; several combinations of conditions also resulted in resting spore production for the Norwegian *N. floridana* isolate in *T. urticae* females, but only at low levels (1.4–5.0%). The majority of the spore-forming conditions (8 out of 9 combinations) included a 10L : 14D light regime. 

The time to lethality in *T. urticae* females varied from 10.0 to 18.0 days for the Norwegian isolate (NCRI271/04) and from 20.0 to 21.9 days for the Brazilian isolate (ESALQ1420) at the temperatures tested. The mites containing resting spores survived longer than the mites with hyphal bodies.

**Figure 1:**
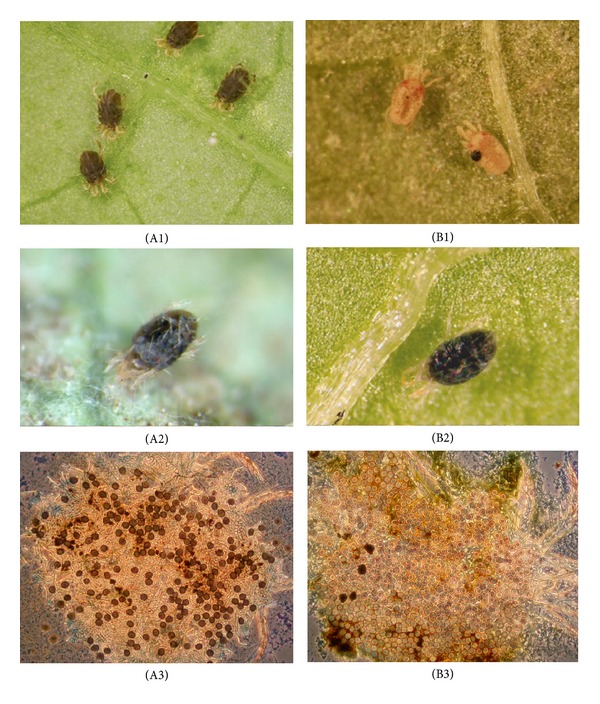
*T. urticae* killed by the fungus *N. floridana*. A(1–3) Norwegian isolate (NCRI271/04), (A1) dark brown cadavers with hyphal bodies, (A2) black/dark brown cadaver with resting spore, (A3) mature resting spores in squash mount, B(1–3) Brazilian isolate (ESALQ1420), (B1) light brown/orange cadavers with hyphal bodies, (B2) black/dark brown cadaver with resting spores, (B3) almost mature resting spores in squash mount.

*T. urticae* cadavers containing resting spores from the Norwegian *N. floridana* isolate (NCRI271/04) were quite different from *T. urticae* cadavers containing resting spores from the Brazilian *N. floridana* isolate (ESALQ1420). Swollen fungus-killed cadavers filled with hyphal bodies, referred to as mummies, were opaque orange/light brown for the Brazilian isolate (Figure 1(B1)) but dark brown/black for the Norwegian isolate (Figure 1(A1)). When *N. floridana* produces resting spore cadavers, *T. urticae* first turns gray/light brown and then shiny dark brown/black and slightly swollen (Figures 1(A2) and 1(B2)). When the resting spores reach maturity, the cuticle of the mite becomes fragile. *T. urticae* cadavers with immature Norwegian *N. floridana* resting spores were of equal size and shape, whereas *T. urticae* cadavers with immature Brazilian *N. floridana* resting spores varied in size and shape (Figures 1(A3) and 1(B3)). The majority of the *T. urticae* cadavers with resting spores also contained hyphal bodies (Figures 1(A3) and 1(B3)).

### 3.2. Experiments Mimicking Tropical Region Conditions

Even though 13,516 *T. urticae* and *T. evansi* (including a pilot experiment, data not shown) were tested under 13 different conditions, no *T. urticae* and a very low percentage of *N. floridana* (ESALQ1421)-killed *T. evansi* adult females (up to 1.0%) produced resting spores under the following conditions: 32°C, RH: 70%, 12L : 12D, and young leaves. Further, 0.5% of *N. floridana* (ESALQ1421)-killed *T. evansi* adult females produced resting spores under the following conditions: 35°C, RH: 60%, 12L : 12D, uninfested leaves. A third condition also resulted in 0.5% resting spore production in *N. floridana* (ESALQ1421)-killed *T. evansi* adult females: 25°C (light period) and 15°C (dark period), RH: 60%, 11L : 13D, and leaves with chlorosis (Tables 2 and 3).

*T. evansi* cadavers containing *N. floridana* isolate ESALQ1421 resting spores were shiny dark brown/black and retained their original mite shape. When the resting spores were mature, the *T. evansi* cuticle became fragile. Further, the mature resting spores of *N. floridana* (ESALQ1421)-killed *T. evansi* were equal in size and shape. *N. floridana* (ESALQ1421)-killed *T. evansi* cadavers with hyphal bodies were distinct from cadavers with resting spores and became swollen and light brown/orange in color.

## 4. Discussion

In this study a small percentage (1.4–5.0%) of *T. urticae* with resting spores was found for the Norwegian *N. floridana* isolate NCRI271/04 under certain temperate region-mimicking conditions. Most of the resting spores produced by the Norwegian isolate (8 out of 9 conditions) were produced under a 10L : 14D light regime and a 16L : 08D light regime. At Ås, in the Southeastern part of Norway, 10 h of light occurs in the fall (17 October) and winter (24 February), and days with 16 h of light occur at the end of the summer (10 August) and in the spring (1 May) (http://www.timeanddate.no/). Our results for the Norwegian *N. floridana* isolate are therefore consistent with earlier field studies in temperate regions that indicated that resting spores of local *N. floridana* isolates in *T. urticae* seem to be induced in fall when the hibernation of *T. urticae* females is also induced [8]. In São Paulo, Brazil, 14 h of darkness never occurs; the shortest day (10 h 40 min) occurs on the winter solstice (21 June). In the experiments mimicking tropical conditions, resting spores were found at very low levels (up to 1.0%) and only in *T. evansi* infected by the Brazilian *N. floridana* isolate ESALQ1421 at high temperatures (32 and 35°C) and a 12L : 12D light regime. In São Paulo, Brazil, days with 12 h of light occur during spring (17 September) and fall (24 March). 

Between-strain differences in the ability to form resting spores have been observed for *Z. radicans* [21] and *E. maimaiga* [26], but this phenomenon has never been investigated in species of *Neozygites* affecting tetranychid mites. The low percentages of resting spores found for the Norwegian isolate in our laboratory studies are similar to the prevalences found in earlier field studies [8] in Norway where resting spore levels in hibernating *T. urticae* females ranged from 2.5 to 13.8%. In these field studies, hibernating *T. urticae* females with hyphal bodies were found at much higher levels, however, and peaked at 54.4%. Our laboratory studies resulting in resting spore infection levels in the Norwegian *N. floridana* isolate of no more than 5.0% at any of the conditions tested may further indicate, as suggested by Klingen et al. [8], that the major overwintering strategy of *N. floridana* in temperate regions is to exist as hyphal bodies inside live hibernating *T. urticae* females and that resting spores are produced mainly for sexual recombination. Other reports have described *N. floridana* resting spores in temperate regions during the autumn and winter, but most of these spores are found at low levels. Klubertanz et al. [6] found resting spores of *Neozygites* sp. in overwintering *T. urticae* in soybeans at a level of approximately 8% of mites sampled. Brandenburg and Kennedy [27] investigated the overwintering strategy of *Entomophthora floridana* (syn. *N. floridana*) in *T. urticae* for two years and observed resting spores in only one sample, collected in autumn (28.0% of mites with resting spores). *T. urticae* with resting spores of *Entomophthora* sp. (syn. *N. floridana*) was observed at some locations in the USA (Clemson, Alabama, Blackville), but no resting spores were found [12]. In temperate regions, *T. urticae* hibernates as adult females [22, 28, 29]. *T. urticae* hibernation is induced by short day length, low temperature, and a lack of nutrition from its host plant [22]. 

In our study, a high percentage (51.5%) of *T. urticae* with *N. floridana* resting spores was found only for the Brazilian isolate ESALQ1420 in the experiments mimicking conditions of temperate regions (11°C, 10L : 14D, and a light intensity of 42–46 *μ*mol m^−2^ s^−1^). These conditions are common in temperate regions but rare in most tropical sites. In tropical regions, it has never been observed that spider mites survive adverse conditions (e.g., drought and lack of host plants) as hibernating females as one may see in temperate regions (e.g., winter) [8]. Further, it is unclear whether resting spore formation is the major strategy for survival under adverse conditions in tropical climates considering that the conditions that best induced resting spore formation for the Brazilian isolate in our experiment are not common in the tropics. 

Resting spores of *Neozygites* sp. have rarely been found in tropical regions. During the nearly 20-year duration of the cassava green mite project investigating *N. tanajoae*, resting spores were found only occasionally in laboratory and field studies. Resting spores of *Neozygites* sp. (= *N. tanajoae*) were observed in northeastern Brazil during the winter [9, 10]. In a field study of *M. tanajoa *and its natural enemy, *N. tanajoae,* Houtondji et al. [30] found only four mites with resting spores in an examination of over 460,000 mites. In more recent studies, however, higher levels of resting spores (34–38%) of *N. tanajoae* in *M. tanajoa* were found by Elliot et al. [9] in northeast Brazil during the winter. The same conditions were tested out in laboratory experiments and also gave high (24.2%) resting spore infection levels. Further, resting spores of *N. floridana* in *T. urticae* were found in southeastern and southern Brazil (Duarte et al. unpublished data; Roggia et al. unpublished data) [4]. These regions have colder winter conditions than those found in northeast Brazil, but the mites with resting spores were found in summer, when the plants become senescent, not in winter when temperatures were as low as in our laboratory experiment. 

The *T. urticae* containing resting spores normally did not die as quickly as *T. urticae* with hyphal bodies. The time to lethality was negatively correlated with temperature. This finding is in accordance with Smitley et al. [31], who found that the mean time to lethality of *T. urticae* infected with *N. floridana* was 15, 5, 4, and 7 days after inoculation when maintained at 10, 20, 30, and 37°C, respectively. Normally, hosts infected by the Brazilian isolate ESALQ1420 die five days after inoculation at 25°C, and those infected with the Norwegian isolate die seven days after inoculation at 20°C (Delalibera Jr. personal communication; Klingen personal communication). 

According to Humber [32] Neozygitales specifically represents the largest and most important “black box” of the new phylum Entomophthoromycota for which needed data remains unavailable. Basic information such as the nature and role of sexual part of the life cycle (resting spore) is still not well characterized. Although we were not able to answer many of our initial questions about resting spore formation and the role of this type of spore, we identified a set of conditions that can consistently produce resting spores, which will be useful for further investigations. 

## Figures and Tables

**Table 1:** Effect of different combinations of photoperiod, mean temperature, temperature drop, light intensity, and light quality on resting spores produced in *N. floridana*-killed* T. urticae* by one isolate from Norway (NCRI271/04) and one from Brazil (ESALQ1420).

Photoperiod	Mean temperature (temperature drop^1^) °C	Host plant conditions	Light intensity (light quality)^2^	Isolate ESALQ1420			Isolate NCRI271/04		
				No. of mites	Hyphal bodies (%)	Resting spores (%)	No. of mites	Hyphal bodies (%)	Resting spores (%)
12L : 12D	25 (−10)	Nonsenescent	165–243 (2)	40	22.5	0	47	17.0	0
	25 (−5)			42	35.7	0	47	25.5	0
	25 (0)			40	30.0	0	46	26.1	0
	25 (5)			40	20.0	0	46	15.2	0
	15		42–46 (1)	60	78.3	0	59	79.7	0

10L : 14D	15 (−10)	Nonsenescent	165–243 (2)	72	50.0	0	69	73.9	0
			247–280 (1)	72	55.6	0	69	66.7	0
			30–35 (2)	72	54.2	0	72	38.9	0
			42–46 (1)	69	68.1	0	72	56.9	**1.4**
		Senescent	165–243 (2)	72	55.6	0	72	59.7	0
			247–280 (1)	71	73.2	0	72	52.8	**2.8**
			30–35 (2)	69	68.1	0	60	56.7	**5.0**
			42–46 (1)	60	81.7	0	63	68.3	**3.2**
	15	Nonsenescent	165–243 (2)	72	51.4	0	69	65.2	0
			247–280 (1)	72	68.1	**1.4**	64	75.0	0
			30–35 (2)	72	75.0	0	72	63.9	0
			42–46 (1)	72	55.6	0	72	50.0	**4.2**
		Senescent	165–243 (2)	72	86.1	0	72	58.3	**1.4**
			247–280 (1)	72	66.7	0	72	69.4	0
			30–35 (2)	69	78.3	0	60	70.0	**1.7 **
			42–46 (1)	72	68.1	0	60	58.3	0
	13	Nonsenescent		60	91.7	0	60	70.0	0
	11		42–46 (1)	111	73.0	**51.5**	102	93.6	**4.7**
	6			60	83.3	0	58	62.1	0

14L : 10D	15	Nonsenescent	42–46 (1)	59	67.8	0	60	78.3	0
16L : 08D				58	93.1	0	59	75.7	**1.7**

^1^Temperature drop: fall of the temperature for 4 h during the light period. ^2^Light intensity (*µ*mol m^−2^ s^−1^) and light quality (1 = warm white fluorescent lamps Philips-Master TL-D 90 and 2 = cool white fluorescent lamps Mitsubishi—40SW (Ra61)).

**Table 2:** Effect of different combinations of abiotic factors (temperature and RH) on resting spores produced in *N. floridana *killed* T. evansi *by two isolates from Brazil (ESALQ1419 and ESALQ1421).

Temperature (°C)	RH (%)	Resting spores (%)	
		ESALQ1421	ESALQ1419
32	60	0	0
	70	1	0
	80	0	0
	90	0	0

35	60	0.5	0
	70	0	0
	80	0	0
	90	0	0

**Table 3:** Effect of different combinations of biotic factors, coinfection, host plant quality, and mite age, on resting spores produced in *N. floridana*-killed* T. urticae* (Brazilian isolate: ESALQ1420) and *T. evansi* (Brazilian isolates: ESALQ1419 and ESALQ1421) isolates from Brazil.

Biotic factors	Treatments	Host	Resting spores (%)
Coinfection	ESALQ1419 × ESALQ1420	*T. evansi *	0
		*T. urticae *	0
	ESALQ1419 × ESALQ1421	*T. evansi *	0

Host plant quality	Chlorosis	ESALQ1420 *T. urticae *	0
		ESALQ1421 *T. evansi *	0.5
	Senescence	ESALQ1421 *T. evansi *	0

Mite age	Larvae	ESALQ1420 *T. urticae *	0
	Adult	ESALQ1420 *T. urticae *	0
